# 79. Hypophosphatasia presenting as seronegative arthritis and mistaken for psoriatic arthritis

**DOI:** 10.1093/rap/rky034.042

**Published:** 2018-09-20

**Authors:** Mia S Rodziewicz, Katie Moss

**Affiliations:** Rheumatology, St George's Hospital, London, UNITED KINGDOM

**Introduction:** Hypophosphatasia (HPP) is a rare metabolic bone disease caused by loss-of-function mutations in the gene encoding tissue-nonspecific alkaline phosphatase (TNSALP). Alkaline phosphatase (ALP) is essential for the normal mineralisation of bone and its reduced function in this disease results in bone pain, fractures, early dental loss and calcium pyrophosphate crystal disease (CPPD) due to accumulation of inorganic pyrophosphate. Disease presentation is heterogeneous, from a perinatal lethal form to milder presentations in adulthood, disease severity correlates inversely with age of symptom onset. HPP is thought to be underdiagnosed in adults in whom there is an estimated prevalence of 1/6370 in Europe. We present a case of a young woman presenting with inflammatory arthritis and a personal history of psoriasis. The diagnosis of HPP was delayed but resulted in the early diagnosis of three of her affected offspring. Afotase alfa is a recombinant ALP and has been recently licenced treatment for adults with childhood onset HPP. This case highlights the importance of careful interpretation of investigations and family history in a patient with seronegative arthritis.

**Case description:** A 27 year old Caucasian woman of Irish descent presented to the rheumatology clinic with a three month history of joint pain and swelling. She had awoken one day with a sudden onset of bilateral painful swollen knees. Three months later she developed pain and swelling of the ankles, wrists, elbows, metacarpalphalangeal (MCP) and proximal interphalangeal joints. She was known to have psoriasis. There was no history of fractures. Her family history was significant for her father having psoriasis and arthritis. She did not take regular medication. Examination revealed puffy, short stubby digits of the hands and nail pitting. There was no clinical evidence of synovitis but she was tender over the wrists, knees and MCP joints. The remainder of the physical examination was normal. Full blood count, urea and electrolytes were within normal ranges. Rheumatoid factor, ACPA and ANA were negative. ESR 25, CRP <1. Bilirubin 7, ALT 10, ALP 23, albumin 45, phosphate 1.35, adjusted calcium 2.37. IgG 18.8, IgA 2.60, IgM 2.2. X-rays of the pelvis, hands and feet showed no evidence of erosions, sacroilitis or chondrocalcinosis. A clinical diagnosis of psoriatic arthritis (PsA) was made and she was commenced on sulfasalazine. She continued to suffer intermittent flares with associated joint swelling that affected the wrists, knees and ankles. She also complained of pain radiating from the knees into the feet. Hydroxychloroquine was added without significant benefit. She was not trialled on any further disease modifying therapies due to several pregnancies. After five years of follow up, the patient then reported difficulty climbing stairs and severe bilateral thigh pain which at times left her unable to walk. A persistently low alkaline phosphatase of below 20 was noted (normal range 30-130 U/L) and a diagnosis of hypophosphatasia was therefore considered. Further questioning revealed that the patient had a delayed walking age of two. Her eldest daughter had 12 teeth extracted at the age of eight due to poor dental health. There was also a family history of premature tooth loss in the maternal side of the family. Further investigations were undertaken. A bone scintigram demonstrated increased uptake in both knees and ankles on delayed imaging. There was no abnormal tracer uptake at the hands or feet. Urinary phosphoethanolamine (PEA) was normal at 9.1 (normal range 4-17.0) μmol/L and serum vitamin B6 was raised at 196 (normal range 35-110) nmol/L. A dual energy x-ray absorptiometry (DEXA) demonstrated normal bone density. Genetic testing confirmed heterogyzoneity for 1 of 280 identified mutations in the TNSALP gene. Further testing of the patient’s offspring confirmed the mutation in three of her five children, representing an autosomal dominant form. It was also later discovered that the patient’s mother had also previously been referred to the rheumatology department with early onset CPPD arthritis at age 20 which responded to colchicine. Our patient was then treated with low dose colchicine 500 mcg bd without significant improvement. Etoricoxib 60 mg was added to hydroxychloroquine 200 mg with only a mild improvement in pain. Six months ago the patient developed synovitis of five MCP joints. She responded well to an intramuscular injection of depomedrone 120 mg and methotrexate 15 mg once weekly was initiated. It was not possible to increase the dose due to nausea and the drug was discontinued after six months due to inefficacy. Leflunomide has recently been commenced.

**Discussion:** In a young person presenting with inflammatory arthritis and a personal and family history of psoriasis, psoriatic arthritis is a probable and common diagnosis. There were however, several features in this case which alluded to another diagnosis. The low serum ALP, late walking age, proximal myalgia, poor dentition and family history of premature tooth loss were all indicators of a diagnosis of HPP. The history early onset CPPD in the patient's mother should have ideally triggered a search for metabolic disorders, including hereditary diseases such as HPP. The patient reported episodes of sudden onset of joint pain and swelling, particularly affecting the knees which was suggestive of a crystal arthritis. These features were not typical of psoriatic arthritis, therefore the diagnosis was re-evaluated. Treatment of HPP involves avoidance of bisphosphonates, calcium and high dose vitamin D supplementation. Patients are advised against contact sports due to increased fracture risk and should receive regular specialist dental follow up. Pseudogout is usually treated by conventional means. Asfotase alfa is human recombinant ALP and was licenced in 2015 for the treatment of hypophosphatasia in adults with childhood onset of severe disease. Unfortunately this patient does not qualify for this treatment despite symptoms which significantly affect her quality of life. Given her personal and family history of psoriasis, it is possible that this patient may have coexisting HPP, CPPD and psoriatic arthritis. It was for this reason that an isotope bone scan was requested to determine the extent and pattern of joint involvement. The symmetrical oligoarticular involvement of large joints suggested that CPPD was the more likely diagnosis at that time. She patient was started on methotrexate due to later evidence of active inflammation of small joints and because this would also be the treatment of choice for PsA. There is evidence that both hydroxychloroquine and methotrexate are of benefit in treating chronic CPPD arthritis. Anti-IL-1 drugs such as Anakinra have been used in small case series for CPPD with some benefit. Aspiration and microscopic evaluation of synovial fluid would have been of great use in confirming CPPD arthritis but still does not exclude dual pathology. This patient has not ever had evidence of a joint effusion on clinical review and therefore synovial fluid analysis has not been possible. A diagnosis of CPPD can be challenging if synovial crystals and chondrocalcinosis on x-ray are absent as in this case. There is emerging evidence of a role for ultrasound and Dual Energy CT scanning in the diagnosis of crystal arthritis, including CPPD. Establishing the correct diagnosis in this patient was particularly valuable as it has allowed genetic screening and early diagnosis and treatment of other family members.

**Key Learning Points:** In a patient with a seronegative non-erosive arthritis, not responding to treatment, the diagnosis should be regularly reviewed. Hypophosphatasia is a rare inherited metabolic bone disease, the milder forms of which can present in adulthood. HPP can present to rheumatology clinic with early onset CPPD arthritis, metatarsal and atypical fractures and/or proximal myopathy. A persistently low serum ALP should prompt detailed evaluation of the dental, musculoskeletal and family history for features of HPP. CPPD arthritis can be challenging to diagnose in the absence of a synovial effusion. There is now effective treatment licensed for adults with childhood onset of severe HPP.

**Disclosure: M.S. Rodziewicz:** None. **K. Moss:** Honoraria; from Alexion for giving lectures.

